# Minimal change disease associated with anti-PD1 immunotherapy: a case report

**DOI:** 10.1186/s12882-018-0958-6

**Published:** 2018-07-03

**Authors:** Bixia Gao, Ningjing Lin, Suxia Wang, Yu Wang

**Affiliations:** 1 Renal Division, Department of Medicine, Peking University First Hospital and Institute of Nephrology, Peking University, Key Laboratory of Renal Disease, Ministry of Health of China, Key Laboratory of Chronic Kidney Disease Prevention and Treatment, Ministry of Education, Beijing, 100034 China; 2 Key Laboratory of Carcinogenesis and Translational Research (Ministry of Education), Department of Lymphoma, Peking University Cancer Hospital & Institute, Beijing, 100142 China; 3 Laboratory of Electron Microscopy, Pathological Centre, Peking University First Hospital, Beijing, 100034 China

**Keywords:** Oncologic immunotherapy, Anti-PD1, Nephrotic syndrome, Minimal change disease

## Abstract

**Background:**

Oncologic immunotherapy is a form of therapy intended to reactivate the immune response to tumor cells using agents that modulate immune checkpoints, such as programmed cell death protein 1 and its ligand (PD-1/PD-L), and cytotoxic T-lymphocyte-associated antigen 4. Along with activation of the immune system to tumors, immune-mediated kidney side effects have been reported, most of which are cases of interstitial nephritis. Glomerular disease, however, appears rare.

**Case presentation:**

Herein, we describe a patient with nephrotic syndrome related to treatment with an anti-PD1 antibody for Hodgkin lymphoma. Following the third dose of anti-PD1 antibody, the patient developed massive proteinuria and nephrotic syndrome. Kidney biopsy showed diffuse podocyte foot process effacement upon electron microscopy, which was consistent with minimal change disease. Corticosteroid treatment yielded full and rapid remission of nephrotic syndrome in 1 month.

**Conclusion:**

The present case suggests an association between anti-PD1 therapeutic immune activation and the development of nephrotic syndrome. Given the increasing prevalence of oncologic immunotherapy, patients should be routinely monitored for kidney side effects associated with these agents.

## Background

Oncologic immunotherapy is being increasingly used for the treatment of both solid and hematologic tumors [1, 2]. With this form of therapy, the immune response to tumor cells is reactivated by modulation of critical immune checkpoint pathways. The programmed cell death protein 1 (PD-1) signaling axis (including its ligand, PD-L) is an established immunotherapeutic target for cancer treatment. However, along with reactivation of the patient’s immune response to tumor cells, immune-related adverse effects (iRAEs) with anti-PD1 therapy have been reported [3, 4]. Kidney side effects related to anti-PD1 therapy are relatively uncommon [5]. Most reported cases presented with acute kidney injury (AKI) induced by interstitial nephritis with predominant tubulointerstitial injury on kidney biopsy [5–7]. Infrequent cases of mass proteinuria and/or nephrotic syndrome (NS) have been reported [8, 9]. Here, we report a patient who developed NS and showed diffuse podocyte foot process effacement consistent with minimal change disease (MCD) during treatment with an anti-PD1 antibody.

## Case presentation

**Fig. 1:**
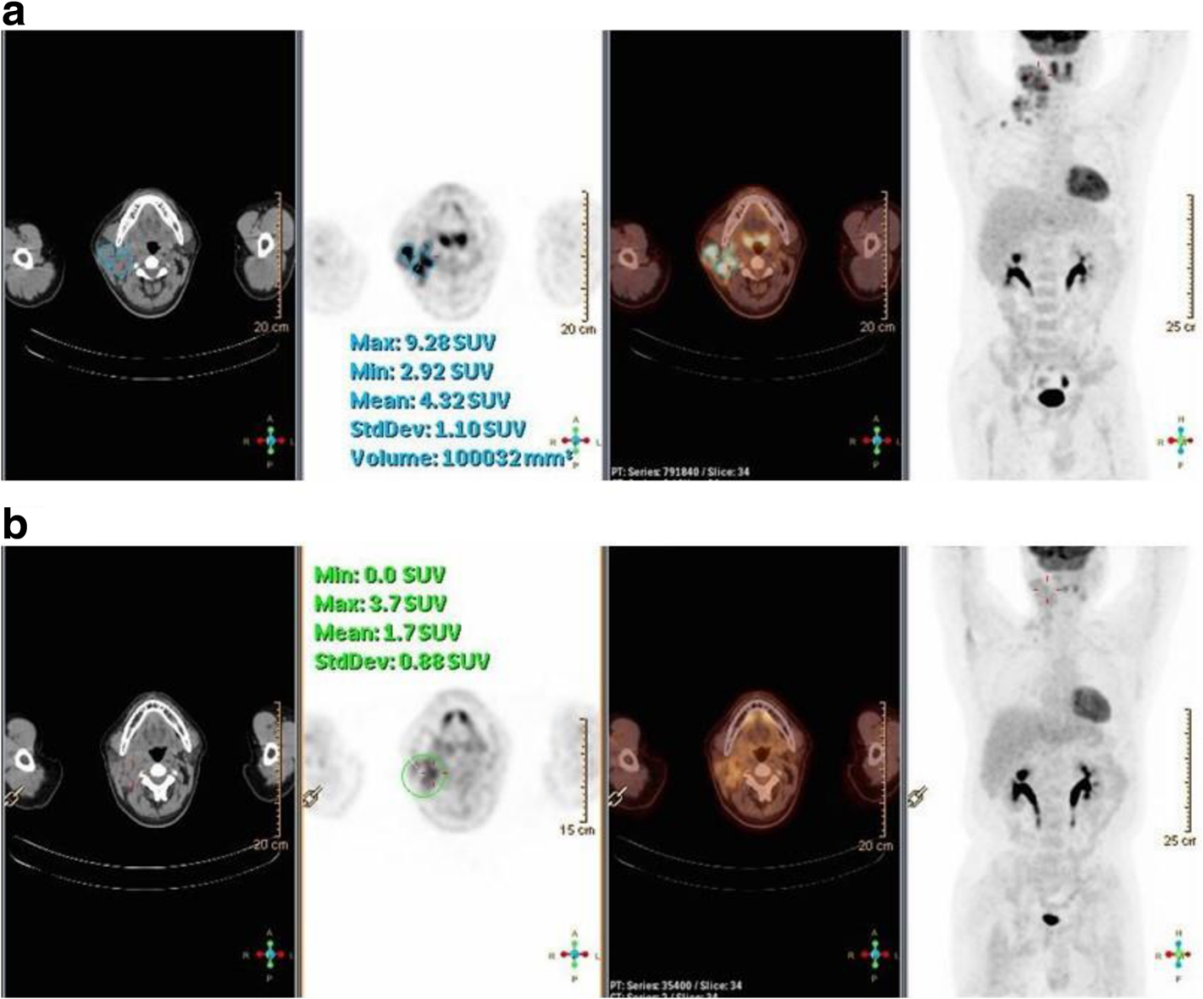
Results of PET/CT scan pre and post anti-PD1 treatment. **a** The images showed hypermetabolic lesions in right cervical, supraclavicular, axillary and interpectoral lymph nodes before anti-PD1 treatment (baseline scan). **b** The images showed the lesions were metabolically less active (score 3 on 5-PS) after 3 cycles of anti-PD1 treatment, which indicated that the patient acquired a complete metabolic response

The 40-year-old male patient was enrolled in a study of anti-PD-1 therapy for Hodgkin lymphoma (HL) after a 3-year history of classical HL that was refractory to classical chemotherapeutic agents. The patient began intravenous administration of an anti-PD-1 antibody (SHR-1210, 200 mg) every 2 weeks. Urine protein was negative prior to the initiation of treatment. After the third dose of the anti-PD1 antibody (30 days from initial treatment), the patient developed massive proteinuria (5.47 g/day) with normal serum albumin and creatinine levels (35.3 g/L and 68 μmol/L, respectively). The treatment was suspended and proteinuria was monitored regularly. His urine protein excretion decreased to 0.47 g/day and further to 0.1 g/day on days 30 and 37, respectively, following the final dose of anti-PD1 antibody. However, 2 weeks later, urine protein excretion increased to 3.21 g/day, and to 30 g/day following an additional 14 days. The patient denied receiving administration of any additional drugs during this period and was admitted for further evaluation. Upon admission, his blood pressure was 110/75 mmHg with moderate pitting edema of both lower limbs. Laboratory tests revealed hypoalbuminemia (21 g/L), normal serum creatinine (80 μmol/L), and elevated total serum cholesterol (6.58 mmol/L). A positron emission tomography/computed tomography scan showed complete metabolic remission of HL (Fig. 1).

**Fig. 2:**
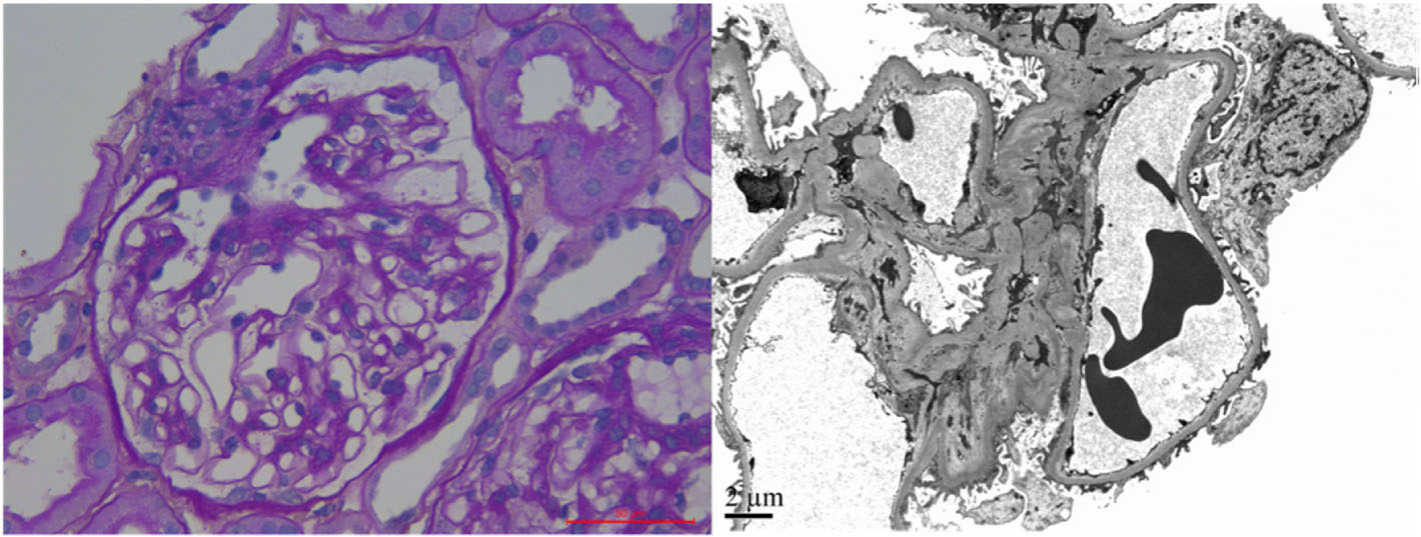
Representative images of kidney biopsy. Left: Light microscopy of the kidney biopsy. Periodic acid-Schiff staining showed glomeruli without obvious change. Right: Representative electron micrograph obtained from kidney biopsy. There was diffuse effacement of foot processes of podocytes

A kidney biopsy was performed. Upon light microscopy, there were 20 glomeruli with no obvious changes. The tubulointerstitium and small arterioles showed no remarkable changes. Immunofluorescence showed the specimen was negative for immunoglobulin G, M, and A, C3, C1q, and κ and λ light chains. Electron microscopy demonstrated diffuse podocyte foot process effacement. The final diagnosis was MCD (Fig. 2). We further screened the secondary causes of MCD. A panel of viral antibodies including hepatitis B virus, hepatitis C virus, human immunodeficiency virus were screened and showed no significant positive results.

**Fig. 3:**
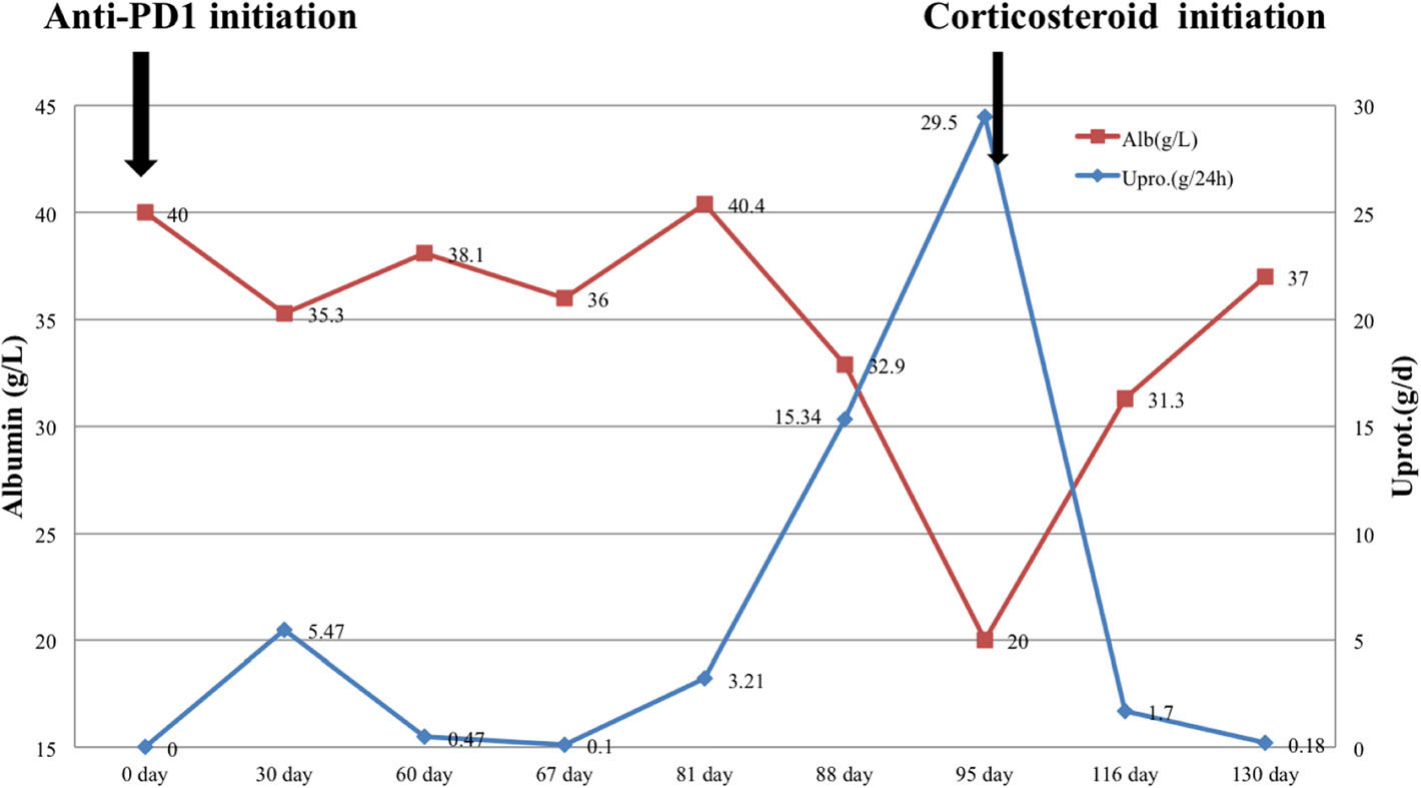
Serum albumin (primary y axis, red squares with trend line) and 24-h urine protein excretion (secondary y axis, blue diamonds with trend line) longitudinally over the anti-PD1 treatment courses

The patient was prescribed prednisone (1 mg/kg/day). Proteinuria improved within 2 weeks (protein excretion decreased to 1.7 g/day, and serum albumin increased to 31.3 g/L). One month following the initiation of prednisone, proteinuria was fully remitted with serum albumin of 37 g/L. Angiotensin-converting enzyme inhibitors and angiotensin receptor blockers were not used. Prednisone was tapered after 8 weeks. Figure 3 shows the changes in serum albumin and 24-h urine protein excretion over the course of treatment.

## Discussion and conclusion

Given the increasing use of immune checkpoint inhibitors (ICPIs) in cancer therapy, nephrologists will be increasingly challenged with diagnosing and managing kidney injury induced by these agents. To date, most reported cases of kidney injury attributed to ICPI therapy were of acute interstitial nephritis with or without concurrent AKI [5–7]. To our knowledge, only one other case of biopsy-proven MCD associated with anti-PD1 immunotherapy, as in the present case, has been described. The previous case also involved the use of an anti-PD1 antibody, pembrolizumab, for the treatment of HL [9]. Kidney biopsy showed MCD with mild acute tubular injury, which resulted in NS with AKI as clinical presentations. Consistent with the patient described herein, the previous case underwent treatment with corticosteroids and achieved full remission of both NS and AKI.

MCD secondary to HL is a well-recognized phenomenon [10, 11]. The temporal relationship between MCD with respect to HL is variable. Therefore, in the present case, whether MCD was caused by anti-PD1 therapy or HL itself remains unclear. Several clinical features suggested that MCD was likely related to anti-PD1 therapy. First, massive proteinuria/NS developed soon after administration of the anti-PD1 agent. Although MCD may develop several months to years following the diagnosis of HL, in the present case, urine protein remained negative during the prior course of HL, and massive proteinuria developed following only three doses of the anti-PD1 antibody, at which point HL entered complete metabolic remission. Second, the patient experienced a period of self-remission of proteinuria after drug discontinuation. This clinical feature was most likely a drug-induced hyperallergic reaction of the kidney, seen primarily with the use of nonsteroidal anti-inflammatory drugs. Third, the patient showed a favorable therapeutic response to prednisone. In a large retrospective study that included 21 adult patients who developed both HL and MCD, patients experienced remission of NS only after successful treatment of HL, regardless of which disease developed first [12]. A similar phenomenon was observed in several other smaller studies [13, 14]. These results indicate that remission of NS requires successful treatment of HL when MCD is a paraneoplastic syndrome in the context of HL. In contrast, ineffective chemotherapy for HL was associated with persistent proteinuria. Our patient entered remission soon after initiation of prednisone treatment for NS. Taken together, these observations suggest MCD in the present case resulted from anti-PD1 therapy.

As one of the major inhibitory receptors that functions as an immune checkpoint, PD-1 may downregulate immune activity by preventing T-cell activation upon binding its ligands (PD-L1 and PD-L2). Therefore, PD-1 may reduce autoimmunity and promote self-tolerance, which protects against overactivation from endogenous or exogenous stimuli under normal physiological conditions. For example, PD-1 knockout mice can develop autoantibody-mediated glomerulonephritis, suggesting that PD-1 plays a role in preventing autoimmune kidney disease [15]. Furthermore, in a murine study of adriamycin nephropathy, blockade of PD-1 worsened kidney histopathological and functional injury, which indicated a protective role of PD-1 [16]. Therefore, as shown in the present report and others, it is plausible that monoclonal antibodies directed against PD-1 can induce adverse immune-related kidney events. In addition to less common kidney events, iRAEs of ICPIs include colitis, pneumonitis, hepatitis, dermatitis, and hypophysitis, which are more common and believed to be autoimmune disorders linked to the expansion of autoreactive T-cells. In most reported cases of ICPI-induced acute interstitial nephritis, extra kidney symptoms often precede or accompany the onset of kidney injury, suggesting a common autoimmune background. It is believed that ICPIs prime or reactivate T-cells with tropism in the kidneys. Although T-cells have also long been suspected to be involved in the pathogenesis of MCD, no experimental or clinical data directly confirm this hypothesis at present. However, given the increasing prevalence of ICPI therapies, monitoring patients for kidney disorders, including both interstitial and glomerular injuries, is warranted.

## Abbreviations

AKI: Acute kidney injury
HL: Hodgkin lymphoma
ICPIs: Immune checkpoint inhibitors
iRAEs: immune-related adverse effects
MCD: Minimal change disease
PD-1: Programmed cell death protein 1
PD-L: Programmed cell death protein ligand
